# P-539. Parainfluenza Virus Serotype-Specific Co-detection with Other Respiratory Viruses and Its Association with Clinical Outcomes in U.S. Children with Acute Respiratory Illness

**DOI:** 10.1093/ofid/ofaf695.754

**Published:** 2026-01-11

**Authors:** Olla R Hamdan, Tess Stopczynski, Justin Z Amarin, Yasmeen Z Qwaider, Haya Hayek, Adam E Gailani, Kalee E Rumfelt, Laura S Stewart, Annabelle de St Maurice, Eileen J Klein, Janet A Englund, John Williams, Marian G Michaels, Peter G Szilagyi, Mary A Staat, Daniel C Payne, Julie A Boom, Leila C Sahni, Jennifer E Schuster, Rangaraj Selvarangan, Ayzsa Tannis, Heidi L Moline, Geoffrey A Weinberg, Andrew J Spieker, James Chappell, Natasha B Halasa

**Affiliations:** Vanderbilt University, Nashville, TN; Vanderbilt University Medical Center, Nashville, Tennessee; Vanderbilt University Medical Center, Nashville, Tennessee; University of Wisconsin, Madison, Wisconsin; Vanderbilt University Medical Center, Nashville, Tennessee; Vanderbilt University Medical Center, Nashville, Tennessee; Vanderbilt University Medical Center, Nashville, Tennessee; Vanderbilt University School of Medicine, Nashville, Tennessee; University of California Los Angeles, Los Angeles, California; Seattle Children's Hospital and University of Washington School of Medicine, Seatte, Washington; Seattle Children’s Hospital/Univ. Washington, Seattle, Washington; University of Wisconsin, Madison, Wisconsin; University of Pittsburgh/ CHP, Pittsburgh, Pennsylvania; UCLA, Los Angeles, California; Cincinnati Children's Hospital Medical Center, Park Hills, Kentucky; Cincinnati Children's Hospital Medical Center, Park Hills, Kentucky; Baylor College of Medicine, Houston, Texas; Baylor College of Medicine and Texas Children's Hospital, Houston, Texas; Children's Mercy Kansas City, Kansas City, MO; Children’s Mercy Hospital, Kansas City, Missouri; Centers for Disease Control and Prevention, Atlanta, Georgia; US-CDC, Atlanta, Georgia; University of Rochester Sch Med & Dent, Rochester, New York; Vanderbilt University Medical Center, Nashville, Tennessee; Vanderbilt University Medical Center, Nashville, Tennessee; Vanderbilt University Medical Center, Nashville, Tennessee

## Abstract

**Background:**

Parainfluenza viruses (PIVs) are a major cause of respiratory illness and hospitalization in children. Human PIVs include four serotypes (1–4), each associated with distinct seasonal patterns and clinical presentations. Data on clinical outcomes of serotype-specific PIV co-detections with other respiratory viruses are limited, potentially masking important distinctions related to PIV diversity.

**Figure 1.**
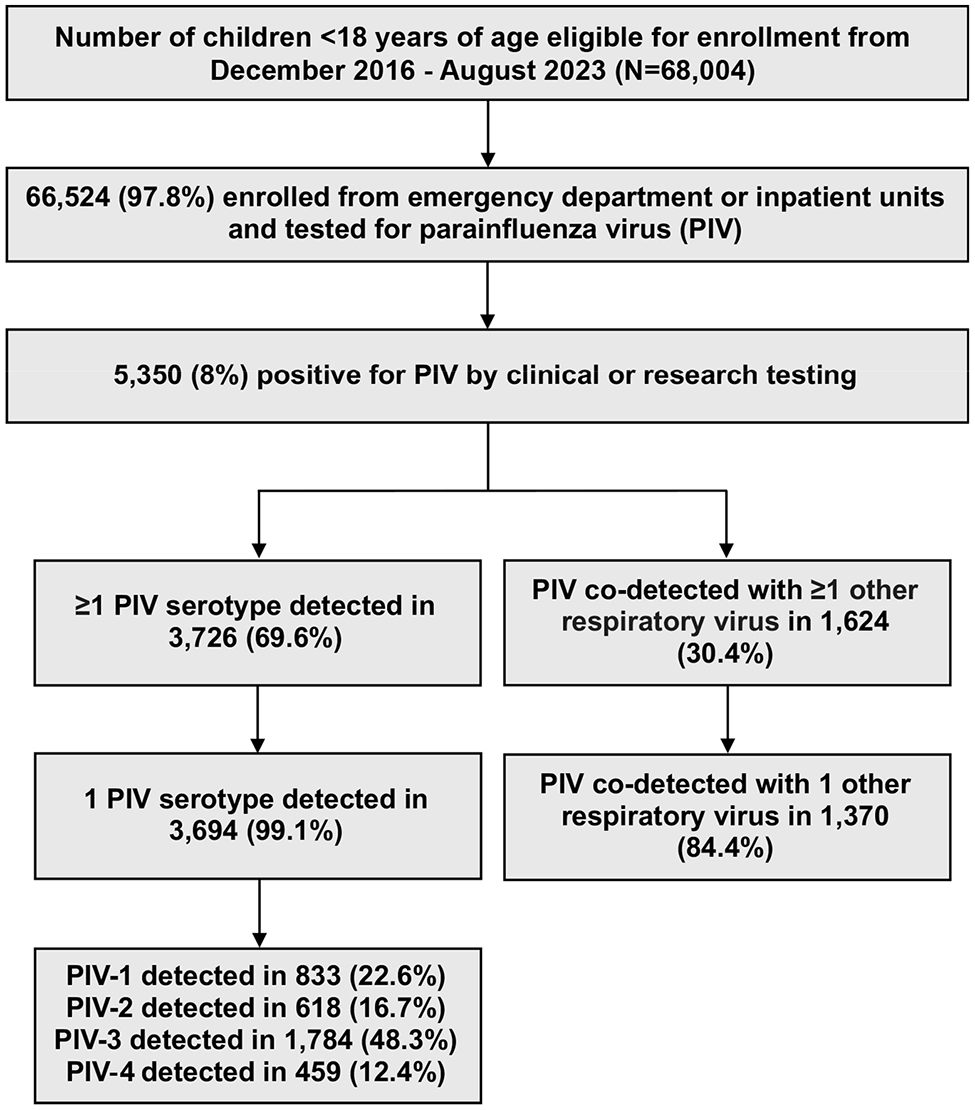
Flow diagram of study participants (December 1, 2016 – August 31, 2023).

**Figure 2.**
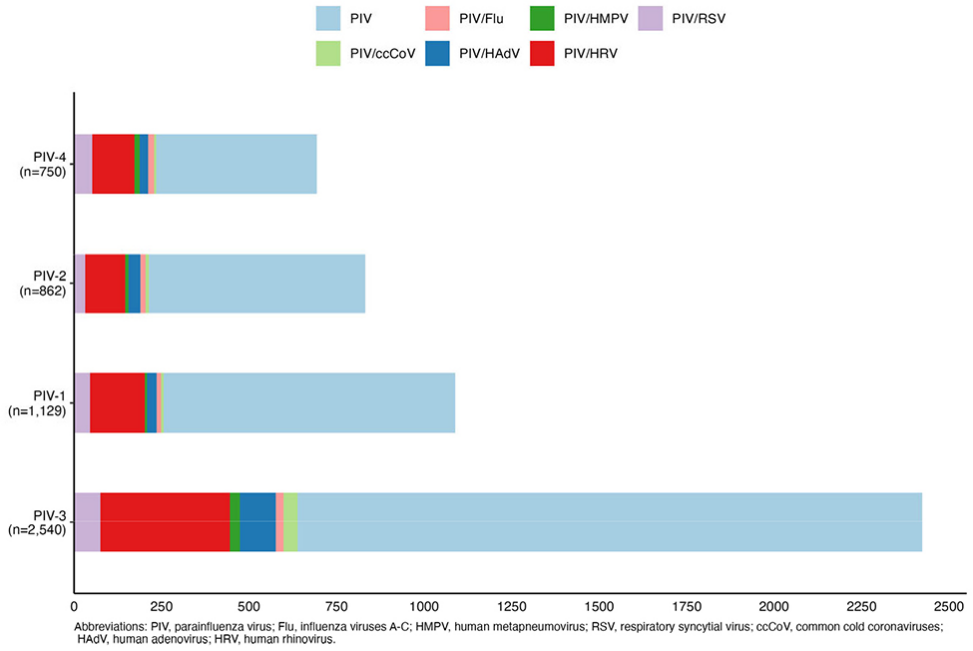
Frequency of children with PIV and only one other respiratory virus detected, stratified by PIV serotype, New Vaccine Surveillance Network (December 1, 2016 – August 31, 2023).

**Methods:**

We conducted active surveillance among U.S. children < 18 years old with medically attended acute respiratory illness at seven centers in the New Vaccine Surveillance Network. Respiratory specimens were tested by RT-PCR for PIV serotypes 1–4 and other common respiratory viruses. We used generalized estimating equations to calculate adjusted odds ratios to compare odds of hospitalization and oxygen use between children with PIV-only detection and the three most commonly co-detected viruses: human rhinovirus (HRV), respiratory syncytial virus (RSV), and human adenovirus (HAdV) accounting for age, prematurity, underlying conditions, site, and clustering by individual.

**Figure 3.**
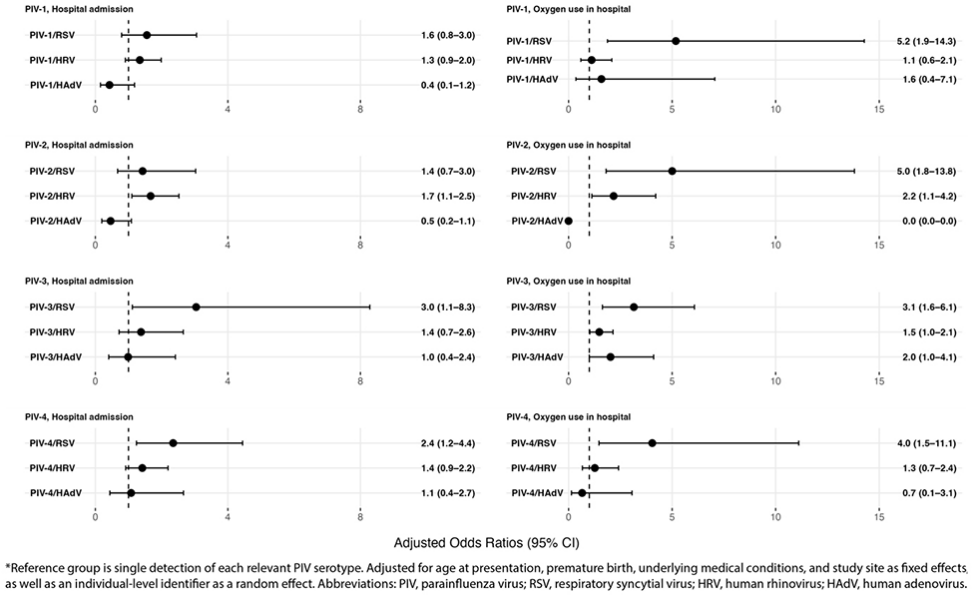
Adjusted odds ratio estimates for hospitalization and inpatient oxygen use in U.S. children with PIV (1–4) only detection and those with PIV (1–4) and one other co-detected respiratory virus, New Vaccine Surveillance Network (December 1, 2016 – August 31, 2023).*

**Results:**

From December 2016 to August 2023, 66,524 children were eligible and enrolled from the emergency department or inpatient units (Figure 1). Of the 5,350 PIV-positive cases, 3,726 (69.6%) had PIV-only detection (including >1 PIV serotype), and 1,624 (30.4%) had at least one additional virus detected with PIV. HRV was most frequently co-detected across PIV serotypes (Figure 2). Compared to PIV-only detection, co-detection of any PIV serotype and RSV was significantly associated with higher odds of oxygen use in the hospital, while PIV-3/RSV and PIV-4/RSV were also associated with higher odds of hospitalization. PIV-2/HRV was associated with higher odds of both hospitalization and oxygen use (Figure 3).

**Conclusion:**

Certain serotype-specific PIV co-detections with HRV and RSV were associated with higher odds of hospitalization or oxygen use. These findings highlight the need to determine PIV serotypes in studies to understand the role of PIV interactions with other respiratory viruses.

**Disclosures:**

Janet A. Englund, MD, AstraZeneca: Board Member|AstraZeneca: Grant/Research Support|Cidarra: Member Data Safety Monitoring Board|GlaxoSmithKline: Advisor/Consultant|GlaxoSmithKline: Grant/Research Support|Meissa Vaccines: Advisor/Consultant|Merck: Advisor/Consultant|Merck: Grant/Research Support|Moderna: Advisor/Consultant|Moderna: Grant/Research Support|Pfizer: Advisor/Consultant|Pfizer: Grant/Research Support|Shionogi: Grant/Research Support Marian G. Michaels, MD, MPH, Merck: Grant/Research Support Mary A. Staat, MD, MPH, Centers for Disease Control and Prevention: Grant/Research Support|Cepheid: Grant/Research Support|Merck: Advisor/Consultant|Merck: Grant/Research Support|National Institutes of Health: Grant/Research Support|Up-To-Date: Royalties Daniel C. Payne, PhD, MSPH, Merck: Advisor/Consultant|Moderna: Advisor/Consultant Rangaraj Selvarangan, PhD, Altona: Grant/Research Support|Biomerieux: Advisor/Consultant|Biomerieux: Grant/Research Support|Biomerieux: Honoraria|Cepheid: Grant/Research Support|Hologic: Grant/Research Support|Hologic: Honoraria|Meridian: Grant/Research Support|Qiagen: Grant/Research Support Geoffrey A. Weinberg, MD, Inhalon Biopharma: Advisor/Consultant|Merck & Co: Honoraria James Chappell, MD, PhD, Merck: Grant support for etiologic studies of acute respiratory illness in hospitalized children, Amman, Jordan Natasha B. Halasa, MD, CSL-Seqirus: Advisor/Consultant|Merck: Grant/Research Support

